# Supplementary material for: Reform influences location of death: Interrupted time-series analysis on older adults and persons with dementia
Source: PLoS One. 2020 Nov 4;15(11):e0241132. doi: 10.1371/journal.pone.0241132 (PMC7641450; doi:10.1371/journal.pone.0241132)
Supplement: S1 Table — (DOCX) [file pone.0241132.s001.docx]

# S1 Table STROBE statement guidelines

STROBE Statement—Checklist of items that should be included in reports of ***cross-sectional studies***

|  | Item No | Recommendation |
| --- | --- | --- |
| **Title and abstract** | 1 | (*a*) Indicate the study’s design with a commonly used term in the title or the abstract Title: Reform influences location of death: Interrupted time-series analysis on older adults and persons with dementia |
|  |  | (*b*) Provide in the abstract an informative and balanced summary of what was done and what was found  **Abstract Methods and Findings:** **This is a repeated cross-sectional, population-level study using mortality data from the Norwegian Cause of Death Registry hosted by the Norwegian Institute of Public Health. Participants were Norwegian older adults 65 years or older with and without dementia who died 2006 to 2017. The intervention was the 2012 Norwegian National Care Reform that increased care infrastructure into communities. The primary outcome was location of death at a nursing home, home, hospital or other location. The trend in the proportion of location of death, before and after the Coordination Reform was estimated using an interrupted time-series analysis. All analyses were adjusted for sex and seasonality. Of the 417,862 older adults identified, 61,940 had dementia identified on their death certificate. Nursing home deaths increased over time while hospital deaths decreased for the total population (Adjusted Relative Risk Ratio (aRRR) 0.87, 95% CI 0.82-0.92) and persons with dementia (aRRR: 0.93, 95%CI 0.91-0.96) after reform implementation.** |
| Introduction | | |
| Background/rationale | 2 | Explain the scientific background and rationale for the investigation being reported  **In 2012, Norway implemented the Coordination Reform as a response to increasing costs, to ensure sustainability of the health care system. Through administrative, structural and economic approaches, tasks and responsibility were transferred from secondary to primary care with a goal to decrease hospitalizations and ensure that services were provided at the lowest efficient care level, closer to the patient’s home. More emphasis was added to providing care to persons at home to allow them to stay longer at home. It is unknown whether place of death was affected.**  **It is also unclear how people with dementia were affected; arguably the most vulnerable and costliest patient population. A majority die in nursing homes. The rationale for institutionalization may differ for persons with dementia compared to persons without dementia because informal caregiver stress in addition to patient characteristics are predictors of institutionalization. There is limited health policy research to determine whether the reform was effective.** |
| Objectives | 3 | State specific objectives, including any prespecified hypotheses  **The aim of this study is to assess the impact of the 2012 Norwegian Coordination Reform on location of death for the total population older than 65 years with and without dementia, based on population-level data. We hypothesize the reform would be associated with a gradual increase in the proportion of people dying in NHs and at home and a decrease in the proportion dying in hospital for persons without dementia. For persons with dementia, we do not expect a difference in the proportions that die in a long-term care setting because of caregiver burden and as this reform involves building infrastructure, more time may be required to see a difference in location of death for this population.** |
| Methods | | |
| Study design | 4 | Present key elements of study design early in the paper  **A population-based observational study** |
| Setting | 5 | Describe the setting, locations, and relevant dates, including periods of recruitment, exposure, follow-up, and data collection  **Death registration data on all deaths for older adult 65 years and older from 2006-2017 in Norway by dementia status** |
| Participants | 6 | (*a*) Give the eligibility criteria, and the sources and methods of selection of participants  **Inclusion criteria comprised:**   - **Decedents aged 65 years or over at time of death** - **Died in Norway between 2006 and 2017 (inclusive) from all causes of death**   **Persons with dementia were identified based on International Classiﬁcation of Diseases, Tenth Revision (codes for dementia: F00.0, F00.1, F00.2, F00.9, F01.0, F01.1, F01.2, F01.3, F01.8, F01.9, F02.0, F02.1, F02.2, F02.3, F02.4, F02.8, F03, G30.0, G30.1, G30.8, G30.9) if dementia diagnosis was included as one of the diagnoses anywhere on the death certificate**  *(b) For matched studies, give matching criteria and number of exposed and unexposed*  **Not applicable secondary analysis** |
| Variables | 7 | Clearly define all outcomes, exposures, predictors, potential confounders, and effect modifiers. Give diagnostic criteria, if applicable  **primary outcome: Place of death as recorded on the death certificate were categorized into home, NH (short-term and long-term), hospital and other (specified).**  **explanatory variables: A time variable (in cumulative quarters) and policy dummy variables indicating the pre-intervention period (coded 0) or the post-intervention period (coded 1) was created. Calendar quarters were included as a categorical variable in the model to account for seasonality. Sex was included as a covariate.** |
| Data sources/ measurement | 8* | For each variable of interest, give sources of data and details of methods of assessment (measurement). Describe comparability of assessment methods if there is more than one group  **All data came from Statistics Norway.** |
| Bias | 9 | Describe any efforts to address potential sources of bias  **Calendar quarters were included as a categorical variable in the model to account for seasonality. We used an equal number of data points before the reform and after the reform.** |
| Study size | 10 | Explain how the study size was arrived at  **All deaths in Norway from 2006 to 2017 meeting the stated inclusion criteria** |
| Quantitative variables | 11 | Explain how quantitative variables were handled in the analyses. If applicable, describe which groupings were chosen and why  **Study** [**population characteristics**](https://www.sciencedirect.com/topics/medicine-and-dentistry/population-and-population-related-phenomena) **and the distribution of place of death were described using unadjusted proportions. Summaries and bivariate comparisons between the outcomes and potential time-varying confounders, and basic before-and-after comparisons were also performed.** |
| Statistical methods | 12 | (*a*) Describe all statistical methods, including those used to control for confounding  **Three weighted multinomial logistic regressions were performed for the total population, persons with dementia and without to calculate** **adjusted relative risk ratios (aRRR) and year-specific mean predicted probabilities of location of death. When calculating predicted probabilities, all other variables were held at their means.** **The regression analyses were weighted to adjust for population growth over the study period. Models included time in cumulative quarters (continuous) since the start of the study, a reform variable (dummy variable), and an interaction term between the reform and cumulative quarters variable. The cumulative quarters variable can be interpreted as the quarterly aRRR of dying in a particular location pre-reform. The reform variable was coded as zero for the pre-reform period, and one for post-reform. The reform variable can be interpreted as the immediate (step) change following the implementation of the reform. The interaction between the cumulative quarters and the reform variables can be interpreted as the quarterly change in relative risk of dying at a particular location since the introduction of the reform (slope change). Calendar quarters were included as a categorical variable in the model to account for seasonality[1]. Sex was included as a covariate.** |
|  |  | (*b*) Describe any methods used to examine subgroups and interactions  **Multinomial logistic regression was performed on the dementia sub group and the non-dementia subgroup. The interaction between the cumulative quarters and the reform variables can be interpreted as the quarterly change in relative risk of dying at a particular location since the introduction of the reform (slope change).** |
|  |  | (*c*) Explain how missing data were addressed **There was no missing data.** |
|  |  | (*d*) If applicable, describe analytical methods taking account of sampling strategy |
|  |  | (*e*) Describe any sensitivity analyses |
| Results | | |
| Participants | 13* | (a) Report numbers of individuals at each stage of study—eg numbers potentially eligible, examined for eligibility, confirmed eligible, included in the study, completing follow-up, and analysed  **Within 2006-2017, 417,862 older adults ≥65 years died. Of these, 61,940 persons were identified with dementia and 355922 people without dementia.** |
|  |  | (b) Give reasons for non-participation at each stage  **Not applicable death registration data** |
|  |  | (c) Consider use of a flow diagram |
| Descriptive data | 14* | (a) Give characteristics of study participants (eg demographic, clinical, social) and information on exposures and potential confounders  **Within 2006-2017, 417,862 older adults ≥65 years died. Of these, 54.3% were female. The pre-intervention proportions for the total population (N=208,667) based on location of death was: NH 48.85%, home 12.44%, hospital 35.28% and other 3.43%, while the post reform proportions (N=209,195) were: NH 54.84%, home 11.84%, hospital 30.10%, and other 3.22%.**  **Altogether, 61,940 persons were identified with dementia; the majority were female (66.29%). The pre-reform location of death (N=27,408) were: NH 86.03%, home 4.57%, hospital 8.15%, and other 1.25%. After the reform (N=34 532), the proportions of location of death were NH 87.95%, home 4.18%, hospital 6.68%, and other 1.18%.**  **For people without dementia (N= 355 922), 52.23% were female. The unadjusted pre-reform proportions for location of death (N=181 259) were: NH 43.23%, home death 13.63%, hospital death 39.38% and other 3.76%. Post reform, the unadjusted proportion (N=174 663) dying at a NH was 48.29%, home 13.35%, hospital 34.73%, and other 3.62%.** |
|  |  | (b) Indicate number of participants with missing data for each variable of interest |
| Outcome data | 15* | Report numbers of outcome events or summary measures  **Main outcome place of death categorized as: hospital, nursing home, home or elsewhere. Nursing home formed the reference group in the multinomial logistic regression verses hospital, home, or other.** |
| Main results | 16 | (*a*) Give unadjusted estimates and, if applicable, confounder-adjusted estimates and their precision (e.g., 95% confidence interval). Make clear which confounders were adjusted for and why they were included  **We used multinomial regression with robust error variance to calculate adjusted relative risk ratios, to investigate whether location of death changed after the Norwegian Coordination Reform was implemented. We used 95% confidence intervals.**  **The regression analyses were weighted to adjust for population growth over the study period. Models included time in cumulative quarters (continuous) since the start of the study, a reform variable (dummy variable), and an interaction term between the reform and cumulative quarters variable. The cumulative quarters variable can be interpreted as the quarterly aRRR of dying in a particular location pre-reform. The reform variable was coded as zero for the pre-reform period, and one for post-reform. The reform variable can be interpreted as the immediate (step) change following the implementation of the reform. The interaction between the cumulative quarters and the reform variables can be interpreted as the quarterly change in relative risk of dying at a particular location since the introduction of the reform (slope change). Calendar quarters were included as a categorical variable in the model to account for seasonality. Sex was included as a covariate.** total population ≥65 years: Before the introduction of the 2012 reform, the proportions of people dying at home (Cumulative quarters, aRRR 0.97, 95% CI 0.96-0.98), hospital (aRRR 0.95, 95% CI 0.95-0.96) and elsewhere (aRRR 0.94, 95% CI 0.93-0.95) were significantly decreasing compared to NHs (Fig 1, S1 Table). After the introduction of the reform, there was evidence of a step change in the proportion of home deaths (aRRR 1.19, 95%CI 1.10-1.29) while the proportion of hospital deaths (aRRR 0.87 95%CI 0.82-0.92) and dying elsewhere (aRRR 0.71, 95%CI 0.62-0.82) decreased compared to NH deaths. The slope change suggests a small but significant deceleration in home deaths (aRRR 0.98, 95%CI 0.96-0.98) and a similarly small but significant acceleration in hospital deaths (aRRR 1.02, 95%CI 1.01-1.02) and dying elsewhere (aRRR 1.06, 95%CI 1.05-1.08) in recent years compared to previous years.persons without dementia: Pre-reform time trends indicated a relative decrease in dying at home (aRRR 0.97, 95% CI 0.97-0.98), at hospital (aRRR 0.96, 95% CI 0.95-0.96) and elsewhere (aRRR 0.94, 95% CI 0.93-0.96) compared to NH for persons without dementia (Fig 1, S1 Table). After the reform, there was no evidence of a step change in dying at home (aRRR 1.08, 95% CI 0.99-1.17), but there was evidence of a significant negative step change for dying at hospital (aRRR 0.83, 95% CI 0.78-0.88) and elsewhere (aRRR 0.65, 95% CI 0.56-0.75) compared to NH deaths. The slope change indicated a small but significant acceleration in the aRRR for hospital deaths (aRRR 1.02, 95% CI 1.01-1.03) and elsewhere (aRRR 1.07, 95% CI 1.05-1.09) in recent years compared to previous years.persons with dementia: Before the introduction of the reform, for persons with dementia there was significant decrease in hospital deaths (aRRR 0.93, 95%CI 0.91-0.96) compared to NH deaths. After the reform, there was evidence of a step change in the proportions of persons with dementia dying at home (aRRR 2.88, 95%CI 2.13-3.90) versus NH deaths. A significant deceleration in home deaths (aRRR 0.90 95% CI 0.86-0.94) and a small but significant acceleration in hospital deaths (1.04 95% CI 1.00-1.08) in recent years compared to previous years was also observed (S1 Table). Results indicate a substantial relative proportional change in hospital deaths, however, in absolute terms, this represents few older adults due to the small population size dying in hospital. |
|  |  | (*b*) Report category boundaries when continuous variables were categorized |
|  |  | (*c*) If relevant, consider translating estimates of relative risk into absolute risk for a meaningful time period |
| Other analyses | 17 | Report other analyses done—eg analyses of subgroups and interactions, and sensitivity analyses |
| Discussion | | |
| Key results | 18 | Summarise key results with reference to study objectives  **The number of people dying in hospital decreased since the 2012 reform for the total population, for persons with and without dementia, while NH deaths increased. Immediately after the reform, home deaths increased for persons with dementia but returned to pre-reform levels over time.** |
| Limitations | 19 | Discuss limitations of the study, taking into account sources of potential bias or imprecision. Discuss both direction and magnitude of any potential bias  **First, using death certificate data that does not provide detailed information regarding changes in places of care closest to death. Second, we relied on the death certificate to identify persons with dementia making these estimates a conservative underestimate. We know that at least 80% of persons in long-term NHs have cognitive impairment. There may be other sociodemographic and health factors that are related to the place of death, but those data were not available.** |
| Interpretation | 20 | **Give a cautious overall interpretation of results considering objectives, limitations, multiplicity of analyses, results from similar studies, and other relevant evidence**  **This study provides evidence that the 2012 Norwegian reform further increased pre-reform trends of less people dying in hospital and that there was an increase in NH deaths. The number of people dying at home has not changed regardless of patient population group.**  **Despite past studies finding death at home being the primary preference [2-4] and policy goals to enable home deaths, we found that there has not been a change in home deaths over time. Interventions to increase support and education in home care services are needed to enable more persons to die at home. Recent work found trajectories of home nursing hours and probability of short-term NH stays indicated possible effective palliative home nursing for some, while others, had not accessed services for staying at home longer at the end-of-life [5]. The authors concluded that continuity of care was an important factor in palliative home care and home death [5].**  **Although home deaths may not have changed, there is evidence that time spent in the community has increased. Previous literature also found length of stay in a long term NH has decreased since the 2012 reform, to a median of 1.31 years in 2016 [6]. One study of 47 NHs within 35 municipalities of Norway (n=691 patients) found 25% of patients died within one year of NH admission [7].** |
| Generalisability | 21 | Discuss the generalisability (external validity) of the study results  **As all datasets used were national registry data, the generalizability of these results are robust. Previous studies have evaluated place of death of people dying from dementia from an international perspective; however, they were cross-sectional and did not focus on countries that have made policy reform [8].** |
| Other information | | |
| Funding | 22 | Give the source of funding and the role of the funders for the present study and, if applicable, for the original study on which the present article is based  **This work was supported by a ZonMw career award, Veni grant # 91619060.** |

*Give information separately for exposed and unexposed groups.

**Note:** An Explanation and Elaboration article discusses each checklist item and gives methodological background and published examples of transparent reporting. The STROBE checklist is best used in conjunction with this article (freely available on the Web sites of PLoS Medicine at http://www.plosmedicine.org/, Annals of Internal Medicine at http://www.annals.org/, and Epidemiology at http://www.epidem.com/). Information on the STROBE Initiative is available at [www.strobe-statement.org](http://www.strobe-statement.org).

**References**

1. Bernal JL, Cummins S, Gasparrini A. Interrupted time series regression for the evaluation of public health interventions: a tutorial. International journal of epidemiology. 2017;46(1):348-55. Epub 2016/06/11. doi: 10.1093/ije/dyw098. PubMed PMID: 27283160; PubMed Central PMCID: PMCPMC5407170.

2. Skår Å JL, Smedslund G, Bahus MK, Pedersen R, Fure B. . Livets sluttfase - om å finne passende behandlingsnivå og behandlingsintensitet for alvorlig syke og døende. Oslo: Nasjonalt kunnskapssenter for helsetjenesten, 2014 Contract No.: Rapport fra Kunnskapssenteret nr. 19−2014. .

3. Gomes B, Higginson IJ, Calanzani N, Cohen J, Deliens L, Daveson BA, et al. Preferences for place of death if faced with advanced cancer: a population survey in England, Flanders, Germany, Italy, the Netherlands, Portugal and Spain. Annals of Oncology. 2012;23(8):2006-15. doi: 10.1093/annonc/mdr602.

4. Faeo SE, Husebo BS, Bruvik FK, Tranvag O. "We live as good a life as we can, in the situation we're in" - the significance of the home as perceived by persons with dementia. BMC Geriatr. 2019;19(1):158. Epub 2019/06/07. doi: 10.1186/s12877-019-1171-6. PubMed PMID: 31170916; PubMed Central PMCID: PMCPMC6555012.

5. Kjellstadli C, Han L, Allore H, Flo E, Husebo BS, Hunskaar S. Associations between home deaths and end-of-life nursing care trajectories for community-dwelling people: a population-based registry study. BMC Health Serv Res. 2019;19(1):698. Epub 2019/10/17. doi: 10.1186/s12913-019-4536-9. PubMed PMID: 31615500; PubMed Central PMCID: PMCPMC6794846.

6. Kjelvik J. Botid i sykehjem og varighet av tjenester til hjemmeboende [Time of residency and services to the community-dwelling]. Trondheim: The Norwegian Directorate of Health; 2017 Report Number 02/2017

7. Sandvik RK, Selbaek G, Bergh S, Aarsland D, Husebo BS. Signs of Imminent Dying and Change in Symptom Intensity During Pharmacological Treatment in Dying Nursing Home Patients: A Prospective Trajectory Study. J Am Med Dir Assoc. 2016;17(9):821-7. Epub 2016/06/21. doi: 10.1016/j.jamda.2016.05.006. PubMed PMID: 27321869.

8. Reyniers T, Deliens L, Pasman HR, Morin L, Addington-Hall J, Frova L, et al. International variation in place of death of older people who died from dementia in 14 European and non-European countries. J Am Med Dir Assoc. 2015;16(2):165-71. Epub 2014/12/30. doi: 10.1016/j.jamda.2014.11.003. PubMed PMID: 25544001.
