# Supplementary material for: Reform influences location of death: Interrupted time-series analysis on older adults and persons with dementia
Source: PLoS One. 2020 Nov 4;15(11):e0241132. doi: 10.1371/journal.pone.0241132 (PMC7641450; doi:10.1371/journal.pone.0241132)
Supplement: S2 Table — aRRR = Adjusted Relative Risk Ratio. Adjusted Relative Risk ratios from three multinomial logistic regression are presented where the reference group is long-term care home. Each regression was adjusted for sex and seasonality. (DOCX) [file pone.0241132.s002.docx]

| S2 Table. Adjusted Relative Risk Ratios from multinomial logistic regressions of location of death for the total population and by dementia status weighted to adjust for population growth over the study period (2012-2017) | | | | | | | | | | | | |
| --- | --- | --- | --- | --- | --- | --- | --- | --- | --- | --- | --- | --- |
|  | Total population | | | | Persons without dementia | | | | Persons with dementia | | | |
|  | N=417,862 | | | | N=355,922 | | | | N=61,940 | | | |
| Outcome  (Reference: Nursing home) | Home death | Hospital | Other | Home death | | Hospital | Other | Home death | | Hospital | Other |  |
| Regression parameters | aRRR  (95% CI)  p-value | aRRR  (95% CI)  p-value | aRRR  (95% CI)  p-value | aRRR  (95% CI)  p-value | | aRRR  (95% CI)  p-value | aRRR  (95% CI)  p-value | aRRR  (95% CI)  p-value | | aRRR  (95% CI)  p-value | aRRR  (95% CI)  p-value |  |
| Fractional year trend  (pre intervention trend) | 0.97  (0.96-0.98)  <0.001 | 0.95  (0.94-0.96)  <0.001 | 0.94  (0.93-0.95)  <0.001 | 0.97  (0.97-0.98)  <0.001 | | 0.96  (0.95-0.96)  <0.001 | 0.94  (0.93-0.96)  <0.001 | 0.97  (0.94-1.00)  0.072 | | 0.93  (0.91-0.96)  <0.001 | 0.99  (0.93-1.06)  0.802 |  |
| 2012  Long  Term  care reform | 1.19  (1.10-1.29)  <0.001 | 0.87  (0.82-0.92)  <0.001 | 0.71  (0.62-0.82)  <0.001 | 1.08  (0.99-1.17)  0.076 | | 0.83  (0.78-0.88)  <0.001 | 0.65  (0.56-0.75)  <0.001 | 2.88  (2.13-3.90)  <0.001 | | 0.83  (0.65-1.06)  0.142 | 1.45  (0.82-2.57)  0.201 |  |
| Reform and fractional year interaction | 0.98  (0.97-0.99)  0.002 | 1.02  (1.01-1.02)  <0.001 | 1.06  (1.03-1.08)  <0.001 | 0.99  (0.98-1.00)  0.299 | | 1.02  (1.01-1.03)  <0.001 | 1.07  (1.05-1.09)  <0.001 | 0.90  (0.86-0.94)  <0.001 | | 1.04  (1.00-1.08)  0.033 | 0.96  (0.88-1.04)  0.297 |  |
| Males | 1.86  (1.82-1.90)  <0.001 | 1.75  (1.73-1.78)  <0.001 | 2.53  (2.44-2.62) <0.001 | 1.81  (1.77-1.85)  <0.001 | | 1.62  (1.59-1.64)  <0.001 | 2.49  (2.40-2.59)  <0.001 | 0.87  (0.80-0.95) 0.002 | | 2.08  (1.96-2.21)  <0.001 | 1.01  (0.87-1.18) 0.858 |  |
| Seasonality* | | | | | | | | | | | |  |
| April-June | 1.00  (0.97-1.03) 0.915 | 1.01  (0.99-1.03) 0.425 | 0.98  (0.93-1.03) 0.463 | 0.99  (0.96-1.02) 0.434 | | 1.00  (0.98-1.02) 0.666 | 0.97  (0.92-1.02) 0.243 | 0.97  (0.87-1.08)  0.578 | | 1.01  (0.92-1.10)  0.871 | 0.99  (0.80-1.21) 0.892 |  |
| July-September | 0.98  (0.95-1.01) 0.124 | 0.99  (0.97-1.01) 0.290 | 1.09  (1.04-1.15) <0.001 | 0.96  (0.93-0.99) 0.008 | | 0.97  (0.95-0.99) 0.004 | 1.08  (1.03-1.13) 0.003 | 0.97  (0.87-1.09)  0.614 | | 1.01  (0.93-1.10)  0.817 | 1.00  (0.81-1.23) 0.983 |  |
| October-December | 1.01  (0.99- 1.04) | 0.98  (0.96-1.00)  0.046 | 1.03  (0.98-1.08)  0.222 | 1.02  (0.99-1.05) 0.239 | | 0.98  (0.96-1.00) 0.114 | 1.03  (0.98-1.08) 0.280 | 1.03  (0.93-1.15) 0.576 | | 1.00  (0.92-1.09)  0.930 | 1.11  (0.91-1.35) 0.296 |  |

*****Reference group for seasonality is January-March)
