# Supplementary material for: Reform influences location of death: Interrupted time-series analysis on older adults and persons with dementia
Source: PLoS One. 2020 Nov 4;15(11):e0241132. doi: 10.1371/journal.pone.0241132 (PMC7641450; doi:10.1371/journal.pone.0241132)
Supplement: Attachment — Submitted filename: Response to reviewers.docx [file pone.0241132.s004.docx]

To the Section Editor of PLOS ONE

Stephen Ginsberg, PhD

Amsterdam, August 21, 2020

Dear Dr. Ginsberg,

Thank you for allowing us to revise our manuscript (Ref. No.: PONE-D-20-19299). We thank the reviewers and Editor for their constructive criticism. Below each comment we start our reply with “**ANSWER**” and in italics we present the changes made in the manuscript along with the page number.

**Response to Reviewer**

**Reviewer #1: “Reform influences location of death: Interrupted time-series analysis on older adults and persons with dementia” examines the effect of a national reform on place of death for the general population and stratified by dementia diagnosis (yes/no). The idea is important, the use of population level data is strong, and the methods are novel. Overall, the study shows that the reform did not move the needle on home death very much, but that an existing trend to push death from the hospital to nursing homes continued after the reform was enacted.**

**Major comments
1. From the introduction, analysis, and discussion, there is not a compelling case that the 2012 reform is a policy cut point that makes sense.**

**ANSWER**: The Coordination Reform was introduced in a white paper [1] in 2009 and municipalities were thoroughly prepared before it was enacted in 2012. In fact, there were economic sanctions in place for municipalities who were unprepared [2].

[page 3, Introduction, line 50-52]

*“After the Coordination Reform was introduced in a white paper in 2009, the Norwegian government implemented it in 2012 as a response to increasing costs, to ensure sustainability of the health care system [1].”*

[page 6, Methods, statistical analysis, line 134-136]

*“Lag variables were not created because the policy was enacted on January 1, 2012 after 3 years of notice. There were economic sanctions for municipalities who were unprepared before January 1, 2012 [1, 4].”*

**Troubling aspects are:
a. Concurrent enactment of other reforms to support end-of-life care in nursing homes seems to undermine the potential impact of the 2012 reform on its own.**

**ANSWER**: We revised our wording to state:

[page 10, Discussion, Main findings, line 183-193]

*“Fewer hospital deaths could be a consequence of the Coordination Reform enabling greater collaboration between NH medicine and palliative care in NHs [22, 23]. After the collaboration, Norwegian NHs were better equipped to handle end-of-life palliative care, resulting in fewer transfers to hospitals in the last weeks of life. Furthermore, there were already trends in a decreased number of deaths in Norwegian hospital pre-reform [13]. A Norwegian study that evaluated location of death in Norway over 25 years (1987–2011 period) found shifts in end-of-life care from hospital to NHs [13]. The authors concluded that this was partly due to policy shifts enabling NHs to provide end-of life care [13]. Our study extends this literature by evaluating the effects of the 2012 reform which appear to have increased the magnitude and reinforced previous policy reform to avoid hospital deaths. Previous studies found transfers to hospitals and death in hospital was negatively associated with quality of life for older adults and persons with dementia [15-18, 24].”*

The article that we cited from Kalseth reported reforms to hospital in 1997. Our approach to evaluate the Coordination Reform remains valid even in the face of other medical, social and policy changes over the time period. We are not attempting to partition all the variation to different factors, rather to associate the Coordination Reform with the outcome. Also, there were no other major health care reforms in the time period we are investigating.

**b. The reform was enacted in 2012, but surely the municipalities were not able to respond to the reform on January 1, 2012? Or was there lead time given to allow municipalities to adjust local resources in response to the reform?**

**ANSWER:** Please see the answer to comment #1.

**c. Provide additional support for why January 2012 is a good—and not just convenient—cut point, or how results may be affected by delayed implementation/response to reforms at regional levels. This is particularly important given:**

**ANSWER:** Please see answer to comment # 1.

**i. Trends observed prior to the 2012 reform appear to have just continued, and in some cases reversed, after the reform.**

**ANSWER:** As we model pre-reform trends, the moment of the reform and after, we can conclude that there are statistical differences after the implementation of the reforms compared to before the reform. The aim of this study is to evaluate changes in location over time; however, a major question when looking at health policy evidence in evaluations is the magnitude of the reform effect [3]. Our results show that there was a small increase in the magnitude of nursing home deaths compared to the preform trend in addition to a decrease in hospital deaths. Location of home deaths did not really change over time. However these reforms can be considered a success as they created societal awareness in advance care planning and there was economic benefit because care was provided closer to home or in a nursing home and avoided stressful end of life hospital admissions [4]. During the SARS-COV-2 we clearly saw the benefits of the Coordination Reform because the nursing homes had previously scaled up the medical staff and had the training to provide end of life care [4]. Our results are important as they provide evidence from the effect of the Coordination Reform over time.

[page 11, Discussion, Health policy implications, line 233-240]

*“Despite our results showing modest change in location of death these reforms can be considered a success as they enabled treatment in place and created societal awareness in advance care planning. Furthermore, there was economic benefit because care was provided closer to home or in a nursing home and avoided stressful end of life hospital admissions [35]. From a clinical perspective, during the SARS-COV-2 outbreak, we clearly saw the benefits of the Coordination Reform because the nursing homes had previously scaled up the medical staff and had the comprehensive training to provide end of life care [35]. These analytics approaches will be valuable for future investigation for the impact of SARS-COV-2.”*

**ii. There were other parallel reforms at/around the same time that could explain the increase in nursing home deaths, which would naturally come from hospital deaths given that is the second most frequent place of death.**

**ANSWER:** Please see answer to comment #1a.

**d. Provide a clearer explanation of what authors can and cannot say about the 2012 reform based on the analysis performed.**

**ANSWER:** For the total population and persons with dementia, it appears the Coordination Reform was associated with an increased aRRR of people dying in the community. This is supported by more people dying in long-term care as well as dying at home and later in the slope change, an increased risk of dying in hospital. We have also added to the limitations section that we present population-level results that are not person specific. The strength of this study is that the results apply to the complete Norwegian population.

[page 11, Discussion, Conclusion, line 242-245]

“*This study provides preliminary evidence at a population-level that the 2012 Norwegian reform enabled treatment in place because of increased older adults having their location of death in a long-term care facility instead of a hospital regardless of dementia status. The number of people dying at home did not change irrespective of patient population group.”*

[page 10, Discussion, Limitations, line 221-222]

*“Third, we present population-level results that are not person-specific; however, the strength of this study is that the results apply to the entire Norwegian population.”*

**2. Explain in the introduction how hospital death is worse for persons with dementia. It is stated in the abstract and cited in the discussion, but not explained or cited in the introduction, making it difficult to understand why the analysis was stratified based on dementia diagnosis.**

**ANSWER:**

[page 3, Introduction, line 75-79]

*“It is also unknown if the Coordination Reform is associated with more persons with dementia dying in hospital. Persons with dementia admitted to hospital are at risk for functional decline, lack of pain control, increased morbidity, increased mortality and a decreased quality of life [15-17]. Furthermore, numerous nursing and medical procedures may be unnecessarily continued or started in the last hours of a patient with dementia’s life [18] and persons with dementia are at high risk for delirium [19].”*

**3. The use and representation of predicted probabilities needs to be clarified. The statistical analysis refers to calculation of predicted probabilities.**

**ANSWER**: After running the multinomial logistic regressions, we calculated the year-specific mean predicted probability of dying in at home, nursing home, hospital and other locations for the total population and by dementia status. We then collapsed the predicted probabilities by year and plotted the values over time.

The revised text reads [page 5, Methods, statistical analysis, line 125-127]:

*Three weighted multinomial logistic regressions were performed for the total population, persons with dementia and without to calculate adjusted relative risk ratios (aRRR) and year-specific mean predicted probabilities of location of death.*

**a. I assume the Figure graphs predicted probabilities but the Y axis units are not labeled and the text refers to “adjusted proportions.” Predicted probabilities are not proportions.**

**ANSWER**: Thank you, we have revised the figure and text to read

*Three weighted multinomial logistic regressions were performed for the total population, persons with dementia and without to calculate adjusted relative risk ratios (aRRR) and year-specific mean predicted probabilities of location of death.*

**b. Clarify how predicted probabilities were calculated: were variables taken at the mean? Mode? Label figure and update text accordingly.**

**ANSWER:** By using the margins command in Stata, we estimated the marginal (e.g. mean over the observed time period) predicted probabilities.

**Minor comments
4. It is unclear how deaths in short- versus long-term nursing homes factor into the analysis. This is mentioned in the hypotheses but not followed up in the analysis**.

**ANSWER**: We deleted this terminology throughout the manuscript.

**5. Reform/Intervention variable is explained twice on page 5 under “explanatory variables” and “statistical analysis.”**

**ANSWER**: This was deleted from the statistical analysis.

**6. For persons not familiar with the methods used, explain what accelerations and decelerations mean. Specifically, do they indicate that any initial effects from the reform were not lasting?**

**ANSWER**: This means that the slope increased or decreased and is now added at the first usage.

[page 8, Results, multinomial logistic regression, line 151-155]:

*This was followed by a small but significant deceleration (e.g. reduced slope) in home deaths (aRRR 0.98, 95%CI 0.96-0.98) and a similarly small but significant acceleration (e.g. increased slope) in hospital deaths (aRRR 1.02, 95%CI 1.01-1.02) and dying elsewhere (aRRR 1.06, 95%CI 1.05-1.08) in recent years compared to previous years.*

**7. The figure is illegible and too wide. Perhaps collapse time points to report on yearly figures as seasonality is adjusted for but not discussed and therefore does not seem that central to the authors’ argument.**

**ANSWER**: We revised the figure

**8. Typo in S2 Appendix: should 2015 reform read 2012 reform?**

**ANSWER**: This has been changed.

**Reviewer #2: A repeated cross-sectional, population-level study using mortality data from the Norwegian Cause of Death Registry using an interrupted time series analysis was appropriate to describe changes in the proportion of older adults with and without dementia dying in nursing homes, home, hospital and other locations since Norway instituted a Coordination Reform. This study was developed using the STROBE and RECORD statement guidelines. The statistical analysis was completed appropriately by including a weighted multinomial logistic regression. All data is available publicly.**

We thank the reviewer for their time to review the manuscript.

**Reviewer #3: The manuscript presented a clear and well-conducted study on an important topic for EOL care. Its methods and analyzes are adequate to the proposed objectives. My suggestions are: reduce the Introduction, leaving the second paragraph for Discussion; and, in the Main findings, a broader comparison with other countries is necessary to allow some generalization of the results. No further inconsistances were found.**

**ANSWER:** We thank the reviewer for their time to review the manuscript. We kindly disagree with moving the second paragraph because it makes the case for the evidence gap. We agree with the reviewer that it would be improve the manuscript to make comparisons with other countries implementing reforms to their long-term care. Unfortunately although reforms are frequently happening to long-term care as a response to the greying of the population, it is rare to make an evaluation of the reform. We rewrote the Health policy implications and generalizability to increase the external validity of the paper and to encourage other countries to create other evidenced-based health policy.

[page 10, Discussion, Health policy implications and generalizability, line 224-240]:

*“This study contributes to society by providing new information on how current strategies have changed end-of-life care for the total population and persons with dementia over time. By evaluating existing care frameworks, we can better understand what is effective based on countries that have actively targeted in-home services supporting older adults to live in the community. In contrast to Norway investing in the community medical infrastructure, the Netherlands implemented austerity measures that included closing down long-term care facilities [35] and cutting homecare by 32% [36]. It is unclear if the Netherlands has overlooked the merits of home care and long-term care facilities, particularly for people with dementia. At an international level, more research is required to evaluate long-term reforms to create evidenced-based health policy. This research may provide a strategic policy roadmap for countries to follow. Despite our results showing modest change in location of death these reforms can be considered a success as they enabled treatment in place and created societal awareness in advance care planning. Furthermore, there was economic benefit because care was provided closer to home or in a nursing home and avoided stressful end of life hospital admissions [37]. From a clinical perspective, during the SARS-COV-2 outbreak, we clearly saw the benefits of the Coordination Reform because the nursing homes had previously scaled up the medical staff and had the comprehensive training to provide end of life care [37]. These approaches will be valuable for future investigation for the impact of SARS-COV-2. As all datasets used were national registry data, the generalizability of these results are robust.”*

References

1.	Ministry of health and care services. Samhandlingsreformen. Rett behandling - på rett sted - til rett tid [The Coordination Reform. Proper treatment – at the right place and right time]. Oslo: Ministry of health and care services, 2009 Report Meld.st.47 (2008-2009).

2.	Forskningsrådet. Evaluering av samhandlingsreformen. Sluttrapport fra styringsgruppen for forskningsbasert følgeevaluering av samhandlinvsreformen (EVASAM). Oslo: 2016.

3.	Baicker K, Chandra A. Evidence-Based Health Policy. New England Journal of Medicine. 2017;377(25):2413-5. doi: 10.1056/NEJMp1709816. PubMed PMID: 29262287.

4.	Husebø BS, Berge LI. Intensive Medicine and Nursing Home Care in Times of SARS CoV-2: A Norwegian Perspective. The American journal of geriatric psychiatry : official journal of the American Association for Geriatric Psychiatry. 2020;28(7):792-3. Epub 04/22. doi: 10.1016/j.jagp.2020.04.016. PubMed PMID: 32381282.
