# Supplementary material for: Reform influences location of death: Interrupted time-series analysis on older adults and persons with dementia
Source: PLoS One. 2020 Nov 4;15(11):e0241132. doi: 10.1371/journal.pone.0241132 (PMC7641450; doi:10.1371/journal.pone.0241132)
Supplement: Attachment — Submitted filename: Response to Reviewers.docx [file pone.0241132.s005.docx]

Dear Editor and the Reviewers,

Thank you for allowing us to revise our manuscript “Reform influences location of death: Interrupted time-series analysis on older adults and persons with dementia*”* (Ref. No.: PONE-D-20-19299R1). We thank the reviewers and Editor for their constructive criticism. Below each comment we start our reply with “**ANSWER**” and in italics we present the changes made in the manuscript along with the page number.

**Response to Reviewers**

Reviewer #1: The authors addressed concerns raised in the initial review by providing additional explanation of the context in which reforms existed and by clarifying analyses. Two minor comments:

1. Clarify in statistical analysis that, when calculating predicted probabilities, all other variables were held at their means.

**ANSWER:** Thank you, we clarified and updated the methods section.

[Statistical Analysis section, page 5, lines:127-128]

"*When calculating predicted probabilities, all other variables were held at their means.”*

2. Change "was" to "were" in the first sentence under "explanatory variables."

**ANSWER**: We corrected the typo.

[Statistical Analysis section, page 5, lines:117-118]

"*A time variable (in cumulative quarters) and policy dummy variables indicating the pre-intervention period (coded 0) or the post-intervention period (coded 1) were created.”*

Reviewer #3:

1. The manuscript explores an important topic in EOL care, and the method and data analysis are clear.
In Results, as seasonality and sex were included as covariate, and nothing else was explained about that, I assume that they didn´t show differences, but it could be briefly declared in the text.

**ANSWER:** We were only using seasonality and sex variables as covariates to answer our main research question and therefore we did not report their results. Guidelines to evaluate healthcare services on location of death [1] recommend including them as variables to be controlled for when evaluating the service impact however they are not the focus of the evaluation. However, we can understand readers may be interested in these variables so we added the seasonality and sex parameter estimates to the table in the appendix. Futhermore, we added text in the results section. For the total population and persons without dementia, males were more likely than females to die at home, in hospital or in another location than a long-term care facility. However males with dementia were less likely than females to die at home and were more likely to die in hospital. Calendar years were not significant in any of the regressions.

[Results section, total population, page 8, lines:156-157]

*“Males were more likely than females to die at home (aRRR 1.86, 95%CI 1.82-1.90), in hospital (aRRR 1.75, 95%CI 1.73-1.78) or in another location (aRRR 2.53, 95%CI 2.44-2.62) than in a NH.”*

[Results section, Persons without dementia, page 8, lines:170-172]

*“Males were more likely than females to die at home (aRRR 1.81, 95%CI 1.77-1.85), in hospital (aRRR 1.62, 95%CI 1.59-1.64) or in another location (aRRR 2.49, 95%CI 2.40-2.59) than in a NH.”*

[Results section, Persons with dementia, page 9, lines:181-183

*“Males with dementia were less likely than females to die at home (aRRR 0.87, 95% CI 0.80-0.95) and were more likely to die in hospital (aRRR 2.08, 95% CI 1.96-2.21).”*

2. I still missed an international approach in Discussion, many countries (in Europe, to be close to the manuscript context) presented a progressive change in place of death from hospital to nursing homes and to home in the last decades, and how much it can be explained by public health policies, epidemiological factors or by wider access to palliative care approach.

**ANSWER:** We have included a new section *“*International comparisons*“*.

[Discussion section, International comparisons, page 10, lines: 220-241]

“**International comparisons**

*A European Commission report [33] that compared reforms to long-term care provisions in 35 countries in the past 10 years (2008-2018) found three overall trends: 1) changes to the long-term care policy mix and shifts from residential care towards home care and community care, 2) improving monetary sustainability and 3) increasing access and affordability of care, including recognizing the importance of informal caregivers. Contrary to European trends, Norway has attempted to increase the quality of care in long-term care to avoid hospital deaths. The UK [34], Belgium [36] and Germany [37] have also tried to shift deaths from hospital to long-term care. Norway like other European countries, is interested in financial sustainability, and has also invested in homecare and the community care.*

*Very few studies evaluate the effects of long-term care reforms on location of death which is surprising considering healthcare resources appear to play a greater role in location of death than individual-level characteristics [38-41]. Gao et al. [34, 39] evaluated the United Kingdom’s National End of Life Care Program [42] aimed to decrease unnecessary emergency admissions, reduce hospital death, improve the skills of the workforce and enable more people to die at the place of their choice [35, 43] and found a decrease in hospital deaths and an increase of home deaths for cancer patients since the care implementation. Gao et al. [40] also proposed a population-level framework to evaluate health services and location of death using health services characteristics and patient-level factors.*

*Location of death for persons with dementia varies across Europe [44]. However, most persons with dementia die in a long-term care facility [44]. To our knowledge, there are no published European studies evaluating the association of national long-term care reforms and location of death for persons with dementia. There have been national plans created in Denmark and Greece targeting care for persons with dementia, but it is unknown if they are associated with a change of location of death [33].”*

3. In particular on Coordination Reform, I would like to ask whether it included specific topics on palliative care in the document, as it is not familiar to most readers.

**ANSWER**: The coordination reform document written in 2008, contained a chapter about the *“*Future tasks and the role of municipalities*”* with a section dedicated to increasing palliative care units in nursing homes. The document listed at the time of publication, 27 palliative units and 164 palliative beds. In 2017 there were 48 palliative units with 294 palliative beds + 147 single palliative beds in Norwegian SNFs.

[Discussion section, Main findings, page 9, lines:192-195]

*“The 2009 Coordination Reform white paper recommended municipalities to increase the number of palliative units in NHs [1]. A report in 2017 found substantial increases in palliative units and beds indicating that the Coordination Reform contributed to palliative care provisions in NHs [24].”*

**References**

1.	Gao W, Huque S, Morgan M, Higginson IJ. A Population-Based Conceptual Framework for Evaluating the Role of Healthcare Services in Place of Death. Healthcare (Basel). 2018;6(3). Epub 2018/09/12. doi: 10.3390/healthcare6030107. PubMed PMID: 30200247; PubMed Central PMCID: PMCPMC6164352.

2.	Det Kongelige Helse-OG Omsorgsdepartment. Samhandlingsreformen Rett behandling – på rett sted – til rett tid 2009. Available from: <https://www.regjeringen.no/contentassets/d4f0e16ad32e4bbd8d8ab5c21445a5dc/no/pdfs/stm200820090047000dddpdfs.pdf>.

3.	Kaasa S, Andersen S, Bahus M, Broen P, Farsund H, Flovik A, et al. På liv og død. Palliasjon til alvorlig syke og døende [On life and death. Palliative care to the seriously ill and dying]. Oslo: Helse- og omsorgsdepartementet, 2017 Report NOU 2017.
